# Short and long-term efficacy of massage for functional constipation: A protocol for systematic review and meta analysis

**DOI:** 10.1097/MD.0000000000020698

**Published:** 2020-06-19

**Authors:** Ying Tang, Kejin Shi, Fengyi He, Mao Li, Yong Wen, Xiaomin Wang, Jie Zhu, Zhao Jin

**Affiliations:** Chengdu University of Traditional Chinese Medicine, Chengdu, Sichuan Province, China.

**Keywords:** functional constipation, massage, protocol, systematic review

## Abstract

**Background:**

Functional constipation (FC) is one of the most common diseases throughout the world, which brings a bad influence on life quality as well as mental health. Massage has been widely used in the treatment of functional constipation in china. In several randomized controlled trials indicate that massage has a positive effect on FC. However, there remain exist controversy towards its effectiveness and safety. What's more, how about the short and long-term efficacy? We, therefore, design this systematic review to assess the short and long-term effects of massage for FC.

**Methods:**

The following electronic databases will be searched from their inception to May 2020, including PubMed, Cochrane Library, EMBASE, Web of Science, WHO International Clinical Trials Registry Platform, Chinese National Knowledge Infrastructure (CNKI), WanFang Database, Chinese Biomedical Literature Database (CBM), the Chongqing VIP Chinese Science, and Technology Periodical Database (VIP).

**Results:**

This systematic review will assess the short and long-term effects of massage in the treatment of FC.

Conclusion: This study will provide high-quality current evidence of short and long-term effects of massage for FC.

**Ethics and dissemination:**

Ethical approval is not required, for this review will not involve individuals’ information. The results will be published in a peer-reviewed publication or disseminated in relevant conferences.

INPLASY Registration number: INPLASY202050001.

## 1 Introduction

Functional constipation (FC) is a common functional gastrointestinal disease (FGID), is characterized by difficulty in defecation, low frequency of defecation, fatigue and sensory insufficiency, but no organic abnormality in the lower abdomen.^[1,2]^ FC is a global public health problem, with a prevalence rate of 6% to 29.6%,^[3]^ which seriously affects patients’ quality of life (QoL),^[4]^ and the medical cost is high.^[5]^ Lifestyle modification and medication are general measures for FC. The curative effect of the former is still uncertain and the recommendation is weak. Laxative treatment is effective, but it usually relapses after withdrawal,^[6]^ and there are side effects such as electrolyte disturbance, dehydration, intestinal cramps, and esophageal obstruction.^[7]^ Therefore, seeking an effective FC replacement therapy with few side effects has attracted the attention of both doctors and patients.

Massage is a safe and non-invasive non-drug treatment with limited contraindications and no known serious side effects.^[8–10]^ In addition, massage can be carried out independently by patients,^[8]^ which can not only increase patients’ participation in treatment, solve the potential psychological factors of constipation,^[11]^ but also reduce the cost of treatment.^[12]^ Clinical trials have confirmed abdominal massage can improve chronic constipation.^[10,13–15]^ Abdominal massage can increase bowel movement, decrease colonic transit time.^[10]^ Previous systematic reviews mainly focused on the efficacy and safety of massage in the treatment of constipation.^[16]^ However, the therapeutic effect of massage may not be apparent until a few weeks later, with a delayed effect,^[15]^ so it is particularly important to evaluate and analyze the short-term and long-term efficacy of its therapeutic effect.

The purpose of this study is:

1. To systematically evaluate the short-term and long-term efficacy of massage for constipation to obtain the duration of its therapeutic effect.
2. Through comparative research with other non-pharmacological therapies, we hope to find out the treatment cycle for the maximum curative effect of massage for constipation.

## 2 Methods

### 2.1 Study registration

The protocol for this systematic review was registered on INPLASY (Unique ID number), and is available in full on the inplasy.com (https://doi.org/10.37766/inplasy00000000). The registration number: INPLASYINPLASY202050001. This systematic review protocol report is based on the PRISMA-P guidelines, and will be conducted in accordance with the PRISMA guidelines.

### 2.2 Inclusion criteria for study selection

#### 2.2.1 Type of study

Randomized controlled trials evaluating massage therapy for FC will be eligible for inclusion and were published in English or Chinese. No publication status restrictions.

#### 2.2.2 Type of participant

Participants aged 18 years or older who diagnosed with functional constipation based on the RomaIII diagnostic criteria will be included. There are no restrictions on gender, nationality, education, or economic status.

#### 2.2.3 Type of intervention

The types of massage including chiropractic therapy, massage using a single thumb, abdominal massage therapy, and spinal manipulation, massage combined with drug therapy will be excluded. The control group interventions including one of the following treatment methods: drugs, acupuncture and moxibustion, cupping therapy, drugs, and physical interventions.

#### 2.2.4 Types of outcome measures

The primary outcome is the frequency of bowel movement (Bowel movement frequency is the mean times per week). The secondary outcomes including quality of life (QoL), mean transit time, patients using laxatives, and adverse event reporting in studies.

### 2.3 Search methods

The following electronic databases will be searched from their inception to May 2020, including PubMed, Cochrane Library, EMBASE, Web of Science, WHO International Clinical Trials Registry Platform, Chinese National Knowledge Infrastructure (CNKI), WanFang Database, Chinese Biomedical Literature Database (CBM), the Chongqing VIP Chinese Science, and Technology Periodical Database (VIP). The search strategy for PubMed is shown in Table 1. Other online databases will be used in the same strategy.

**Table 1:**
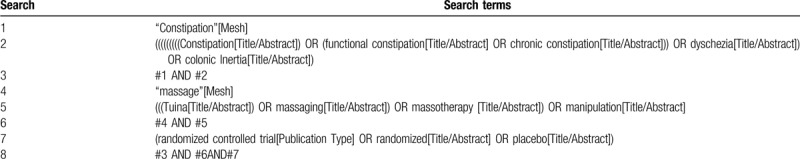
Search strategy used in PubMed.

### 2.4 Data collection and analysis

#### 2.4.1 Selection of studies

**Figure 1:**
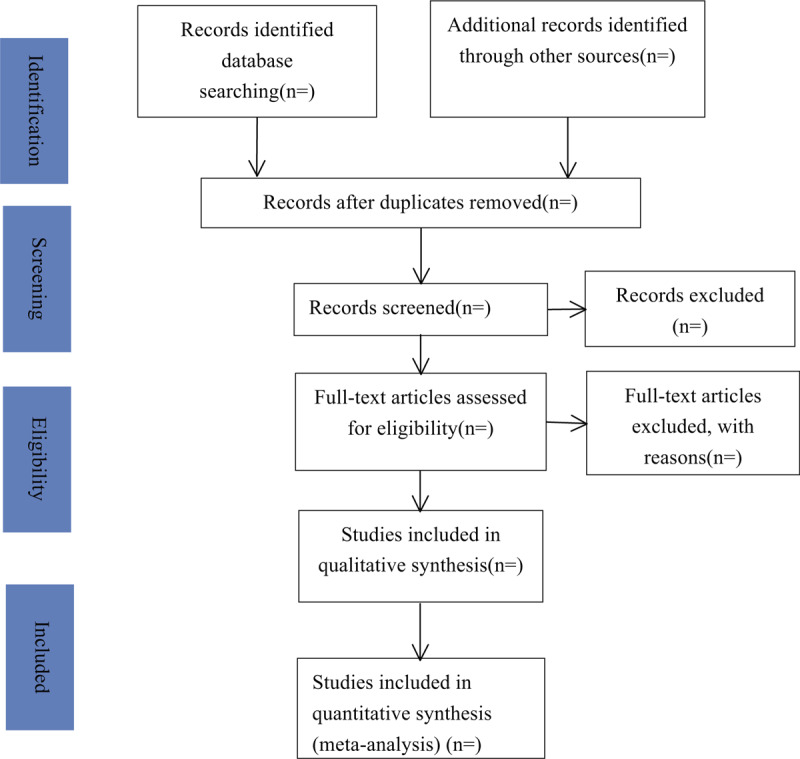
Flow chart of study selection process.

Two authors will independently select the trials according to the inclusion criteria, and import into Endnote X9. Then remove duplicated or ineligible studies. Screen the titles, abstracts, and full texts of all literature to identify eligible studies. Obtain the full literature of all eligible trials. The third reviewer will resolve the selection divergence. The selection process is performed in a PRISMA flow chart (Fig. 1)

#### 2.4.2 Data collection and management

Two independent reviewers will extract data from the included literature: name of the first author, published date, age, gender, and sample size, type of control, main outcomes, and additional outcomes, side effects, follow-up time. Any disagreement will be determined by negotiation with the third reviewer. Then we will use the Review Manager Software (RevMan V.5.3.5) analysis and synthesize data.

#### 2.4.3 Assessment of risk of bias in included studies

Two authors will independently evaluate the risk of bias based on the Cochrane Collaboration's tool. The bias will be conducted into three levels: “low risk”, “high risk,” and “unclear” according to the following criteria: sequence generation, blinding, allocation concealment, incomplete data, selective outcome reporting, and other sources of bias. Any opposition caused by assessment of the study bias, there is a need for determining by the third reviewer.

#### 2.4.4 Measures of treatment effect

Two authors will independently finish the data analysis. Risk ratio (RR) with 95% confidence interval will be applied for dichotomous data. Standard mean difference or standard mean difference with 95% CI will be applied for continuous outcomes. RR form will be changed to analyze other binary data.

#### 2.4.5 Unit of analysis

The analysis unit will be the individual participant.

#### 2.4.6 Management of missing data

The reviewer will contact the corresponding author for the details if basic information is missing. If the missing data is still not available, all data from known results will be conducted an available case study.

#### 2.4.7 Assessment of heterogeneity

The heterogeneity of the study will be evaluated by Q-test and I^2^ statistics. The interpretation of I^2^ is follows: indicates homogeneous (0%–40%), moderate heterogeneity (30%–60%), substantial heterogeneity (50%–90%), considerable heterogeneity (75%–100%). Meta-regression method will be adopted to analyze the cause of the heterogeneity when I^2^ is more than 50%.

#### 2.4.8 Assessment of reporting biases

We will use the Egger test to detect the symmetry of funnel plots to assess the reported biases if available studies more than 10 trials, and Egger will be used to investigate the symmetry of funnel plots.

#### 2.4.9 Data synthesis

RevManV.5.3.5 will be applied for data synthesis. The fixed-effect model will be used for data synthesis if heterogeneity is low, while the random-effects model will be adopted if the heterogeneity is moderate. But if there is significant heterogeneity, we will perform the subgroup analysis or descriptive analysis, or the narrative and a qualitative summary.

#### 2.4.10 Subgroup analysis and investigation of heterogeneity

Subgroups will be performed in accordance with follow up time (1 month, 1–3 months, more than 3 months). If the previous analysis suggests considerable heterogeneity, we will perform a further subgroup analysis.

#### 2.4.11 Sensitivity analysis

Sensitivity analysis will be applied to explore the robustness and reliability of the results. The sample size, studies design, methodological quality, and missing data will be assessed. Then, we will analyze the data again after the exclusion of low methodological quality trials.

#### 2.4.12 Summary of evidence

The quality of evidence will be evaluated according to grading of recommendations assessment, development, and evaluation (GRADE), and will be adjudicated into high, moderate, low, and very low quality.

## 3 Discussion

FC is a common gastrointestinal disease, which seriously affects the patient's quality of life and increases the patient's medical burden. Although laxative is effective, they are not necessarily suitable for all patients, leading to some side effects. As a supplementary and alternative therapy, massage has been widely used in clinical practice. Professionals in the field of massage therapy continue to recommend abdominal massage for constipation.^[17]^ Although massage increasing popularity as a choice of non-pharmacological treatment nowadays, the mechanism has not been clearly established.

According to traditional Chinese medicine (TCM), massage on the epidermis of the body can stimulate and adjust the distribution of meridians, qi and blood, affect the overall function of the internal organs, and adjust the gastrointestinal movement, which can achieve a good effect on the treatment of constipation.^[18]^ Previous studies have suggested that abdominal massage promotes defecation not only through activation of intestinal stretch receptors, but also by stimulating somatic-autonomic reflex to produce rectal waves.^[19]^ Abdominal massage may also stimulate the parasympathetic nervous system, thereby reducing abdominal muscle tension, increasing digestive tract muscle exercise capacity, increasing digestive tract secretions, and relaxing digestive sphincter muscles to promote bowel movements.^[17,20]^ In addition, research shows that, abdominal massage requires a certain period of time to influence constipation.^[21]^ How long abdominal massage should be administered is also an important question.

Therefore, we will analyze previous randomized controlled trials to determine its short and long-term of massage in the treatment of FC. To our knowledge, this study will be the first systematic review of this issue. This review may have some limitations. We believe that the result of this systematic review will provide valuable information for health authorities, policymakers, and physicians.

## Author contributions

**Conceptualization**: Ying Tang.

**Data curation**: Kejin Shi, Fengyi He.

**Formal analysis**: Mao Li.

**Investigation**: Ying Tang

**Methodology**: Ying Tang.

**Project administration**: Ying Tang, Jie Zhu.

**Software**: Xiaomin Wang, Yong Wen.

**Supervision**: Zhao Jin.

**Writing – original draft**: Ying Tang,Kejin Shi,

**Writing – review & editing**: Ying Tang.
